# Default Mode Network Connectivity as a Function of Familial and Environmental Risk for Psychotic Disorder

**DOI:** 10.1371/journal.pone.0120030

**Published:** 2015-03-19

**Authors:** Sanne C. T. Peeters, Vincent van de Ven, Ed H. B. M Gronenschild, Ameera X. Patel, Petra Habets, Rainer Goebel, Jim van Os, Machteld Marcelis, Genetic Risk and Outcome of Psychosis (G.R.O.U.P.)

**Affiliations:** 1 Dept. of Psychiatry and Psychology, School for Mental Health and Neuroscience, EURON, Maastricht University Medical Center, Maastricht, The Netherlands; 2 Dept. of Cognitive Neuroscience, Faculty of Psychology and Neuroscience, University of Maastricht, Maastricht, The Netherlands; 3 Behavioral and Clinical Neuroscience Institute, Department of Psychiatry, University of Cambridge, Cambridge, United Kingdom; 4 King's College London, King's Health Partners, Department of Psychosis Studies Institute of Psychiatry, London, United Kingdom; 5 Institute for Mental Health Care Eindhoven (GGzE), Eindhoven, The Netherlands; Institution of Automation, CAS, CHINA

## Abstract

**Background:**

Research suggests that altered interregional connectivity in specific networks, such as the default mode network (DMN), is associated with cognitive and psychotic symptoms in schizophrenia. In addition, frontal and limbic connectivity alterations have been associated with trauma, drug use and urban upbringing, though these environmental exposures have never been examined in relation to DMN functional connectivity in psychotic disorder.

**Methods:**

Resting-state functional MRI scans were obtained from 73 patients with psychotic disorder, 83 non-psychotic siblings of patients with psychotic disorder and 72 healthy controls. Posterior cingulate cortex (PCC) seed-based correlation analysis was used to estimate functional connectivity within the DMN. DMN functional connectivity was examined in relation to group (familial risk), group × environmental exposure (to cannabis, developmental trauma and urbanicity) and symptomatology.

**Results:**

There was a significant association between group and PCC connectivity with the inferior parietal lobule (IPL), the precuneus (PCu) and the medial prefrontal cortex (MPFC). Compared to controls, patients and siblings had increased PCC connectivity with the IPL, PCu and MPFC. In the IPL and PCu, the functional connectivity of siblings was intermediate to that of controls and patients. No significant associations were found between DMN connectivity and (subclinical) psychotic/cognitive symptoms. In addition, there were no significant interactions between group and environmental exposures in the model of PCC functional connectivity.

**Discussion:**

Increased functional connectivity in individuals with (increased risk for) psychotic disorder may reflect trait-related network alterations. The within-network “connectivity at rest” intermediate phenotype was not associated with (subclinical) psychotic or cognitive symptoms. The association between familial risk and DMN connectivity was not conditional on environmental exposure.

## Introduction

The disconnection hypothesis postulates that both cognitive and pathophysiological alterations contribute to dysfunctional integration of a distributed network of brain regions in schizophrenia [1,2]. Dysfunctional integration is often addressed with the concept of functional connectivity, which refers to the temporal correlation between two (or more) spatially distinct brain regions [3]. Functional connectivity can be examined in diverse networks. The default mode network (DMN) is active during rest and deactivated when goal-directed behavior is required and is thought to play a role in appraising external and internal stimuli, self-referential and reflective processes. Regions representing the DMN consist of the medial prefrontal cortex (MPFC), the posterior cingulate cortex (PCC) extending into the precuneus (PCu), the lateral parietal cortices, lateral temporal cortex, hippocampus (HC) and parahippocampal gyrus (PHG) [4,5]. Structural and functional alterations in these regions have been associated with schizophrenia [6]. In addition, the DMN has been implicated in self-referential processing [7,8], perspective-taking, self-other judgments [9,10], processing of agency [11] and memory functions [12], all of which appear to be altered in individuals with psychotic disorder. Misinterpretations in some of these processes may contribute to the formation of positive symptoms [8,13].

Studies on DMN connectivity in schizophrenia have shown conflicting results as to the direction of associations. Both decreased, increased and mixed patterns of functional connectivity [14–20], or no significant alterations in patients with schizophrenia [21] have been reported. Similarly, in individuals at higher than average risk for psychotic disorder (first-degree relatives) both increased (in the MPFC, bilateral inferior temporal gyrus (ITG), PCu) [22–24], and reduced functional connectivity (in prefrontal areas, PCC, PCu, ITG) [20,25,26] as well as an absence of significant differences with respect to controls [16,21] have been reported. Taken together, most studies have shown increased connectivity in patients with schizophrenia and first-degree relatives, though the larger studies (n = 258 and n = 799) suggest that patients have reduced DMN connectivity and that relatives have reduced [20] or no differences [16] in DMN connectivity with respect to controls.

(Subclinical) psychotic experiences [27] may arise from impaired monitoring or attribution of agency, which has been associated with posterior lateral parts of the DMN [11]. DMN resting-state studies using seed-based correlation analysis found that increased connectivity between the PCC and respectively the MPFC, other PCC regions, and temporal lobe areas including language areas [22,28] as well as decreased connectivity between the PCC and the temporal gyrus was associated with positive symptoms [28]. Moreover, two resting-state studies using independent component analysis (ICA) found that increased medial and superior frontal gyrus connectivity and decreased hippocampal and inferior parietal cortex connectivity was associated with positive symptoms [1,29]. Areas of the DMN have also been implicated in cognitive functions such as social cognition [7] and working memory (WM) capacities [12,30], and alterations therein have been associated with (the vulnerability for) schizophrenia [7,31–33]. As DMN activity is suppressed during cognitive tasks, altered connectivity in rest may lead to compromised suppression and decreased cognitive performance [1]. Indeed, studies have shown that DMN hyperconnectivity in patients with schizophrenia and their first-degree relatives was associated with reduced WM performance [22]. The relation between social cognition (e.g., Theory of Mind) and DMN resting-state functional connectivity in psychotic disorder has not been examined thus far.

Altered DMN connectivity may not only be conditional on genetic risk for psychotic disorder, but also on established environmental risk factors for schizophrenia such as cannabis use [34], childhood trauma [35] and developmental urbanicity [36]. To date, no resting-state functional connectivity studies have examined gene-environment interaction (G×E) in psychotic disorder. Nevertheless, resting-state fMRI studies have shown that altered stress-anticipation in individuals with a history of childhood poverty [37] and posttraumatic stress disorder (based on early life stress) [38] was associated with reduced DMN connectivity (PCC, PCu, PFC), whereas a task-based fMRI study in individuals showed a positive association between urban upbringing and perigenual anterior cingulate cortex (pACC) activity [39]. In addition, chronic cannabis use has been associated with altered resting-state PCC connectivity, but only in subjects without psychopathology [40].

The current study tested three hypotheses. First, it was hypothesized that individuals with (increased risk for) psychotic disorder would reveal aberrant connectivity (decreased and increased) within the DMN compared to healthy controls. Second, altered DMN connectivity, especially between the PCC and MPFC, in individuals with (increased risk for) psychotic disorder was expected to be associated with positive symptoms (i.e., hallucinations and delusions) and decreased (social) cognitive functioning. Third, it was examined whether DMN functional connectivity reflects a cerebral phenotype that is the outcome of G×E interaction in psychotic disorder.

## Methods

### Participants

Data pertain to baseline measurements of a longitudinal MRI study in Maastricht, the Netherlands. For recruitment and inclusion criteria of patients, their siblings and healthy controls, see [41].

The original sample comprised 89 patients with psychotic disorder, 97 siblings of patients with psychotic disorder and 88 controls. Forty-six participants were excluded from the analyses based on: high schizotypy (n = 3), movement (n = 8) or scanner artifacts (n = 14), smoking cannabis prior to scanning (n = 1) and experimental issues (n = 20). This resulted in a final sample comprising 73 patients with psychotic disorder, 83 siblings of patients with psychotic disorder and 72 controls. The sample comprised 46 families: 25 families with one patient and one sibling, three families with one patient and two siblings. One family with two patients, six families with two siblings, and two families with one patient and three siblings. In the control group, there were nine families with two siblings. In addition, 41 independent patients, 34 independent siblings, and 54 independent controls were included.

Diagnosis was based on the Diagnostic and Statistical Manual of Mental Disorder-IV (DSM-IV) criteria [42], assessed with the Comprehensive Assessment of Symptoms and History (CASH) interview [43]. Patients were diagnosed with: schizophrenia (n = 47), schizoaffective disorder (n = 9), schizophreniform disorder (n = 4), brief psychotic disorder (n = 2), and psychotic disorder not otherwise specified (n = 11). The CASH was also used to confirm the absence of a diagnosis of nonaffective psychosis in the siblings and absence of a lifetime diagnosis of any psychotic disorder or current affective disorder in the healthy controls. The occurrence of any psychotic disorder in first-degree family members also constituted an exclusion criterion for the controls. Schizotypy was assessed with the Structured Interview for Schizotypy-revised (SIS-r) [44]. Ten controls and 16 siblings were diagnosed (lifetime) with major depressive disorder, but none of them presented in a current depressive state.

Before MRI acquisition, participants were screened for the following exclusion criteria: 1) brain injury with unconsciousness of greater than 1 hour, 2) meningitis or other neurological diseases that might have affected brain structure or function, 3) cardiac arrhythmia requiring medical treatment, and 4) severe claustrophobia. In addition, participants with metal corpora aliena were excluded from the study, as were women with intrauterine device status and (suspected) pregnancy.

### Ethics statement

The standing ethics committee of Maastricht University approved the study, and all the participants gave written informed consent in accordance with the committee’s guidelines and with the Declaration of Helsinki [38]. All participants understood the information given to them and could make an informed decision, which was verified by an experienced psychologist. Therefore, all participants included in the study were able to give informed consent without the use of a legal representative or guardian.

### Behavioral Measures

Psychotic symptom assessment was carried out using the Positive and Negative Syndrome Scale (PANSS) [45]. The five factor model by van der Gaag (2006) was used, dividing the PANSS in positive symptoms, negative symptoms, disorganization symptoms, excitement, and emotional distress [46].

Theory of Mind (ToM) was assessed using the raw scores of the hinting task. This is a simple ToM test in which the participants must infer the intention behind indirect speech. The task has a maximum score of 20 [47].

WM was assessed using the raw scores of the arithmetic test of the Wechsler Adult Intelligence Scale-III (WAIS-III) [48]. This test consists of 20 timed arithmetic problems that address verbal comprehension and arithmetic skills.

Educational level was defined as highest accomplished level of education. Handedness was assessed using the Annett Handedness Scale [49].

Antipsychotic (AP) medication use was determined by patient report and verified with the treating consultant psychiatrist. Best estimate lifetime (cumulative) AP use was determined by multiplying the number of days of AP use with the corresponding haloperidol equivalents and summing these scores for all periods of AP use (including the exposure period between baseline assessment for the G.R.O.U.P. study and the moment of baseline MRI scanning), using the converting formulas for AP dose equivalents described in Andreasen and colleagues [50].

### Substance use

Substance use was measured with the Composite International Diagnostic Interview (CIDI) sections B, J and L [51]. Use of cannabis and other drugs was based on the lifetime number of instances of drug use. CIDI frequency data on lifetime cannabis use were available for 220 participants (4% missing data). In addition, cannabis was tested in urine (18% missing data). The two measures were combined into one variable, which was coded as follows: never used cannabis = 0, ever used cannabis = 1 (0% missing data). Data on other drug use were available for 223 participants (2% missing data). Data on cigarette smoking and alcohol use were available for 212 participants (7% missing data) and 206 participants (9% missing data), respectively.

### Childhood trauma

Childhood trauma was assessed with the Dutch version of the Childhood Trauma Questionnaire Short Form (CTQ). The short CTQ consists of 25 items rated on a 5-point Likert scale (1 = never true to 5 = very often true) inquiring about traumatic experiences in childhood. Five types of childhood maltreatment were assessed: emotional, physical and sexual abuse and emotional and physical neglect, with five questions covering each type of trauma [52]. A general measure of childhood trauma was created by calculating the mean of the 25 items. The CTQ data were missing for two participants (1% missing data).

### Level of developmental urbanicity

A historical population density record for each municipality was generated from 1930 onwards using the Dutch Central Bureau of Statistics (CBS) and equivalent Belgium database [53]. It was determined where the subject lived at birth, between ages 0–4 years; between 5–9 years; 10–14 years; 15–19 years; 20–39 years; 40–59 years; and 60+ up to the actual age. For each of these records, the average population density was computed (by square kilometer, excluding water) of the municipality. Average population density was categorized in accordance with the Dutch CBS urbanicity rating (1=<500/km^2^; 2 = 500–1000/km^2^; 3 = 1000–1500/km^2^; 4 = 1500–2500/km^2^; 5 = 2500+/km^2^). The periods 0–4 years, 5–9 years and 10–14 years were merged to average urbanicity exposure between 0–14 years. The latter was used as the primary variable reflecting developmental urbanicity exposure in the analyses. This variable was collapsed *a priori* into 5 intervals (1 to 1.49 = 1; 1.5 to 2.49 = 2; 2.5 to 3.49 = 3; 3.5 to 4.49 = 4; 4.5 to 5 = 5) to reflect the same categories as used by the Dutch CBS [53]. Data on developmental urbanicity were available for all participants (0% missing data).

### MRI acquisition

Functional and anatomical MRI images were acquired using a 3T Siemens scanner. The functional resting-state data were acquired using an Echo-Planar Imaging (EPI) sequence: number of volumes: 200; TE: 30 ms; TR: 1500 ms; voxel size: 3.5x3.5x4.0 mm^3^; flip angle 90°; total acquisition time: 5 min. During the scan, participants were instructed to lie with their eyes closed, think of nothing in particular, and not fall asleep. In addition, anatomical MRI scans had the following acquisition parameters: (1) Modified Driven Equilibrium Fourier Transform (MDEFT) sequence: number of slices: 176; voxel size: 1 mm isotropic; TE: 2.4 ms; TR: 7.92 ms; inversion time: 910 ms; flip angle: 15°; total acquisition time: 12 min 51 s; (2) Magnetization Prepared Rapid Acquisition Gradient-Echo (MPRAGE; Alzheimer’s Disease Neuroimaging Initiative) sequence: number of slices: 192; voxel size: 1 mm isotropic; TE: 2.6 ms; TR: 2250 ms; inversion time: 900 ms; flip angle 9°, total acquisition time: 7 min 23 s. For both anatomical scans the matrix size was 256x256 and field of view was 256x256 mm^2^. Two sequences were used because of a scanner update during data collection. The MPRAGE and MDEFT are very similar, but to prevent any systematic bias, the total proportion of MPRAGE scans (44%) was balanced between the groups.

### Data preprocessing and analysis

Imaging data were preprocessed to account for head motion, as described by Patel et al. (2014) [54] and Jo et al. (2013) [55] using Analysis of Functional NeuroImages (AFNI, version 2011_12_21_1014) [56] as well as the Oxford Centre for Functional MRI of the Brain Software Library (FSL, version 5.0.4) [57,58]. The first four volumes of each resting-state data set were removed to eliminate the non-equilibrium effects of magnetization. Preprocessing steps included slice-time correction, motion correction, despiking of the functional data (removing artifactual outliers in voxelwise time series), temporal bandpass filtering (0.02–0.1 Hz), co-registration to structural scan, spatial normalization to standard space and spatial smoothing (6-mm full width at half maximum Gaussian kernel). Several sources of spurious variance (nuisance variables) were removed from the data through linear regression: six motion correction parameters and their first temporal derivatives, and cerebrospinal fluid (CSF) signal from ventricular regions of interest.

#### Functional connectivity analysis

BrainVoyager QX [59] and routines in Matlab (The Mathworks, Natick, MA, U.S.A.) were used (NeuroElf toolbox [www.neuroelf.net] and custom routines) to estimate functional connectivity for each participant using seed-based correlation analysis. First, whole brain signal intensity averaged across all brain voxels and white matter signal (derived from extracting the BOLD time course signal from a manually defined white matter ROI) were removed from the resting-state data via linear regression. Then, a correlation map was computed using an initiating seed region with a 6-mm radius in the posterior cingulate (PCC, MNI coordinate: 1, -55, 17) based on a previously described method [60]. In the current analysis the PCC was chosen as seed in a seed-based correlation analysis because of its central role in functions of the DMN (e.g. self-referential mental thoughts, WM). Furthermore, it is suggested that the PCC is the only region in the DMN that directly interacts with all other regions within this network [61] and has the highest metabolic activity compared to all other regions during rest [5,62].

Pearson’s correlation coefficients were computed between the time courses of the PCC seed and all other brain voxels and normalized using the Fisher’s r-to-z transformation. Visualization of group effects was restricted to those voxels that empirically were associated with the DMN in all participants. For this purpose, we created a DMN mask by thresholding a one-sample t-test map of the PCC connectivity across all participants, using a false-discovery rate FDR of q = 0.05 [63]. We then performed an ANCOVA with group as between-subject factor, controlling for the subject-level confounders sex, age, handedness and level of education. Significant group effects were visualized using a statistical (p = 0.05, uncorrected) and cluster-size threshold (52 voxels (i.e., 1404 mm^3^)). The cluster-size threshold was estimated using a simulation procedure that incorporates the spatial smoothness of the statistical map (1,000 Monte Carlo simulations [59,64]). The simulated maps were thresholded at the same voxel threshold as the statistical map and surviving clusters were tabulated. The minimum cluster size was selected by taking a false positive rate of 5%.

#### Group differences in DMN connectivity

As selection of regions with a significant between-subject (group) effect was performed using a voxel-level ANCOVA, which assumes independency of groups. Post-hoc analyses on mean individual functional connectivity coefficients of the voxel clusters were performed using multiple linear regression analyses in STATA (corrected for the same confounders) [65]. This was done using the REGRESS command in STATA with regional functional connectivity measures as dependent variables and group as independent variable. Group was entered as dummy variables (controls = 0, siblings = 1, patients = 2). Because of the non-independency of the groups (familial relationships) analyses were repeated with a multilevel random regression model using the XTREG command in STATA. In addition, the voxel-level ANCOVA and subsequent post-hoc tests were performed with full correction for the subject-level confounders tobacco, alcohol, cannabis and other drugs, as DMN connectivity may be influenced by these substances [66–70]. Lastly, since aberrant (increased and decreased) DMN connectivity has also been found in patients with major depression [71], a priori planned sensitivity analyses were carried out excluding all individuals with a history of affective disorder.

#### Associations between DMN connectivity and psychopathology ratings

The associations between DMN functional connectivity (independent variable) and (subclinical) positive symptoms / (social) cognitive performance (dependent variable) were examined with multiple linear regression analyses on the ANCOVA selected regions with a significant between-subject (group) effect. In patients, the association between DMN functional connectivity and symptoms was corrected for age, sex, lifetime AP medication and illness duration (analyses of PANSS positive symptoms). In siblings and controls, the subclinical symptom analyses were corrected for group, age and sex and the (social) cognition analyses were additionally corrected for handedness and level of education. Associations with (social) cognitive performance were investigated in the combined group (of patients, siblings and controls) and corrected for group, age, sex, handedness and level of education.

In order to examine whether the association between DMN functional connectivity and subclinical positive symptoms / (social) cognitive performance would be conditional on group, interactions were tested between group and DMN connectivity, in the ANCOVA selected regions. In case of significant interactions, stratified effect sizes for DMN connectivity were calculated for each group by using the STATA MARGINS routine. Analyses with subclinical positive symptoms were corrected for age and sex, whereas (social) cognitive performance was corrected for age, sex, handedness and level of education.

#### Associations between environmental exposure and DMN connectivity

Main effects of the three a priori hypothesized environmental exposures (cannabis, childhood trauma and developmental urbanicity) on functional connectivity were examined with multiple linear regression analyses using the ANCOVA selected regions with a significant between-subject (group) effect. The environmental exposures were entered as linear variables and as dummy variables (never or ever used cannabis; the childhood trauma score divided by its tertiles (low, medium, high trauma scores); five levels of population density).

In order to study whether altered DMN functional connectivity was the outcome of differential sensitivity to these environmental exposures, two-way interactions between group and environmental exposure (GxE) were examined and evaluated by Wald test [72]. In case of significant interactions, stratified effect sizes for all levels of environmental exposure per group were calculated by linear combination of effects from the model containing the interactions using the STATA MARGINS routine. Analyses were adjusted for the a priori hypothesized confounders age, sex, handedness and level of education.

In addition, associations between AP medication and functional connectivity were analyzed in patients only, with AP medication as independent variable and age, sex and illness duration as confounders.

To control for type I error, significant p-values were subjected to correction for multiple testing using the Simes method [73]. The Simes method avoids overcorrection associated with the Bonferroni correction in case the statistical tests are not independent, as was the case in the present study.

## Results

### Participant characteristics

There were more men than women in the patient group, whereas the opposite held for the control group. Patients had lower educational level than controls and siblings. Patients smoked more cigarettes and used more cannabis and other drugs (lifetime) than siblings and controls. Siblings used more alcohol than patients and controls. Patients, being in stable remission, had relatively low PANSS scores, and performed worse on WM and ToM indices compared to siblings and controls. Childhood trauma was more frequently experienced in patients than in siblings and controls, with no differences between the latter two. The three groups did not differ in developmental level of urban upbringing (Table 1). Out of 73 patients, 64 used AP medication at the time of scanning (second generation: n = 60; first generation: n = 4). The mean current dosage of AP medication in terms of standard haloperidol equivalents was 5.3 mg (SD = 4.8 mg). Furthermore, twelve patients used antidepressants, three used benzodiazepines, five used anticonvulsants and one used lithium. Two siblings and two controls used antidepressants, and one control used benzodiazepines.

**Table 1:** Participant characteristics.

	Patients (n = 73)	Siblings (n = 83)	Controls (n = 72)
	mean (SD)	mean (SD)	mean (SD)
**Age at scan**	27.8 (6.6)	29.6 (9.1)	30.0 (10.8)
**Sex n(%) male**	49 (65%)	45 (54%)	26 (36%)
**Handedness**	72.1 (63.9)	80.1 (53.8)	73.5 (61.2)
**Level of education**	4.2 (2.0)	5.2 (1.9)	5.4 (1.8)
**Cannabis use^1^**	51.7 (47.6)	18.1 (36.0)	8.4 (22.8)
**Cigarettes use^2^**	11.4 (11.0)	2.6 (6.2)	1.9 (6.1)
**Alcohol use^3^**	6.7 (13.0)	10.1 (17.7)	5.1 (7.2)
**Other drug use^4^**	44.4 (87.5)	6.4 (33.0)	2.4 (12.8)
**PANSS positive**	9.7 (4.1)	7.4 (1.5)	7.3 (1.2)
**PANSS negative**	11.9 (6.0)	8.5 (2.2)	8.2 (1.0)
**PANSS disorganization**	12.0 (3.3)	10.4 (1.0)	10.2 (1.2)
**PANSS excitement**	9.9 (2.9)	8.6 (1.4)	8.3 (1.1)
**PANSS emotional distress**	12.7 (5.1)	9.9 (2.7)	9.3 (2.1)
**SIS-r positive subscale**		0.6 (0.4)	0.5 (0.5)
**Hinting task (social cognition)**	18.0 (2.9)	19.2 (1.3)	19.3 (1.1)
**WAIS-III Arithmetic (WM)**	12.5 (4.2)	15.5 (3.7)	15.5 (4.1)
**CTQ total**	7.3 (2.9)	5.8 (1.6)	5.6 (1.8)
**Level of developmental urbanicity**	2.3 (1.3)	2.3 (1.4)	2.6 (1.5)
**Age of onset (yrs)**	21.4 (6.8)		
**Illness duration (yrs)**	6.4 (3.7)		
**Lifetime exposure to AP^5^**	7022.9 (6711.3)		

Abbreviations: SD = standard deviation, PANSS = Positive and Negative Syndrome Scale; SIS-r = Structured Interview for Schizotypy-revised; WAIS = Wechsler Adult Intelligence Scale; WM = Working Memory; CTQ = Childhood Trauma Questionnaire; AP = Anti-Psychotics.

(^1^) Lifetime number of instances of cannabis use

(^2^) Number of daily consumptions over the last 12 months

(^3^) Number of weekly consumptions over the last 12 months

(^4^) Lifetime number of times of hard drug use

(^5^) Lifetime number of days of AP use

### Group differences in DMN connectivity

**Fig 1:**
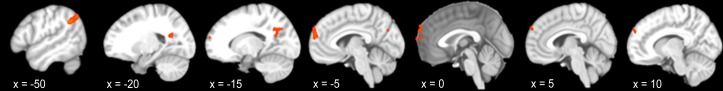
Areas showing significant between-subject (group) effect in PCC connectivity. Results from voxel-level ANCOVA analysis.

**Fig 2:**
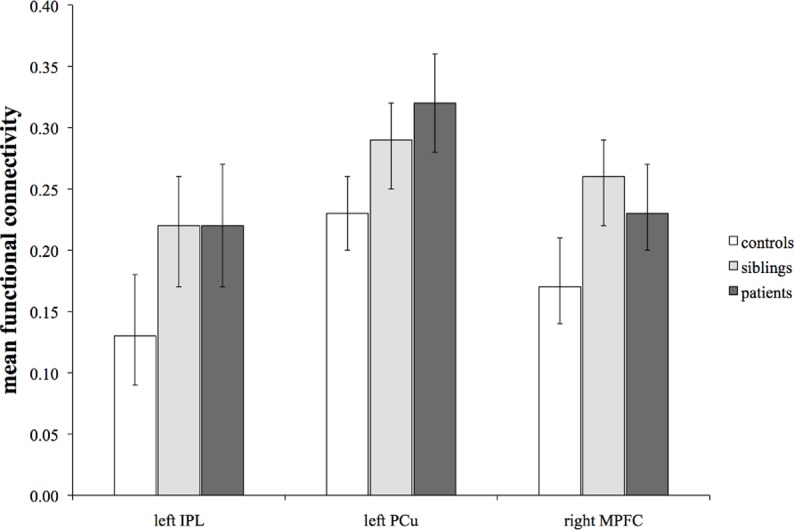
Mean functional connectivity with 95% confidence interval for each region of the DMN that showed significant differences between the groups. There was significantly higher PCC connectivity with the left IPL, left PCu and right MPFC in siblings and patients than in controls, with no significant differences between patients and siblings.

In all three groups, the PCC connectivity map showed significant positive correlations with other DMN regions, including medial frontal, lateral prefrontal, parietal and temporal areas (i.e., the hippocampal complex). The voxel-level ANCOVA revealed a between-subject (group) effect in three DMN regions: the left inferior parietal lobule (IPL), the left precuneus (PCu) and the right medial prefrontal cortex (MPFC) (Fig. 1, Table 2). Post-hoc analyses revealed that patients and siblings had increased connectivity between the PCC seed and left IPL, left PCu and right MPFC (Fig. 2, Table 3). No significant differences were observed between patients and siblings. Multilevel random regression analyses with XTREG did not influence the results. All significant findings were upheld after Simes correction (p_Simes:_ p<0.033) (Table 3). Repeating the voxel-level ANCOVA and post-hoc analyses with additional confounders (tobacco, alcohol, cannabis and other drugs) did not affect the pattern of results, as did the exclusion of siblings and controls with a history of affective disorder (S1 and S2 Table).

**Table 2:** Regions of the default mode network with a significant between-subject (group) effect.

Anatomical region	Hemisphere	Peak coordinates (MNI)			Cluster size (voxels)
		x	y	z	
Inferior parietal lobule	L	-48	-64	43	82
Precuneus	L	-15	-59	33	63
Medial prefrontal cortex	R	9	56	31	89

Results from voxel-level ANCOVA analysis. Voxel size equals 3x3x3 mm^3^. Results represent regions with significant group differences using a statistical threshold p = 0.05 (uncorrected) and cluster threshold (52 voxels). Abbreviations: R, right; L, left.

**Table 3:** Associations between familial risk of psychotic disorder (group) and functional connectivity.

Regions of Interest	Functional Connectivity N = 228			Group differences in functional connectivitymultiple linear regression analyses						Group differences in functional connectivitymultilevel random regression analyses					
	Patients	Siblings	Controls	P vs. C		S vs. C		P vs. S		P vs. C		S vs. C		P vs. S	
	mean (SD)	mean (SD)	mean (SD)	B	p	B	p	B	p	B	p	B	p	B	p
**Left inferior parietal lobule**	0.22 (0.21)	0.22 (0.22)	0.13 (0.21)	0.13	0.001*	0.10	0.004*	0.03	0.385	0.13	0.000*	0.10	0.003*	0.03	0.377
**Left precuneus**	0.32 (0.16)	0.29 (0.16)	0.23 (0.13)	0.10	0.000*	0.07	0.007*	0.03	0.161	0.10	0.000*	0.07	0.003*	0.03	0.180
**Right medial prefrontal cortex**	0.23 (0.15)	0.26 (0.17)	0.17 (0.14)	0.08	0.004*	0.09	0.000*	-0.01	0.593	0.08	0.003*	0.09	0.000*	-0.01	0.586

The Bs represent the regression coefficients from multiple linear regression and multilevel random regression analyses in STATA corrected for age, sex, handedness and level of education. Abbreviations: P = patients; S = siblings, C = controls; SD = standard deviation; the asterisks

(*) represent areas which are significant after Simes correction (P_Simes_<0.033).

### Association between DMN connectivity and positive symptoms

There was no significant association between PANSS positive symptoms and DMN connectivity in the patients (left IPL: B = 1.37, P = 0.630; left PCu: B = -0.83, P = 0.829 and right MPFC: B = 0.54, P = 0.889). Repeating the analyses in patients with the 50% highest positive symptom scores (mean score = 12.24, SD = 4.36, range: 8 to 24) did not change the results. In the combined sibling and control group, SIS-r positive subscale scores and DMN functional connectivity were not significantly associated (left IPL: B = -0.02, P = 0.926; left PCu: B = 0.11, P = 0.637 and right MPFC: B = 0.02, P = 0.946). In addition, no significant group×DMN connectivity interactions in the model of subclinical positive symptoms were found (left IPL (F = 0.69, P = 0.408), left PCu (F = 0.01, P = 0.909) and right MPFC (F = 0.15, P = 0.699)).

### Association between DMN connectivity and cognitive symptoms

In the total group, there was a significant association between WM performance and PCC connectivity with the left PCu (B = -3.63, P = 0.033), but not with the left IPL (B = -1.22, P = 0.309) and right MPFC (B = -0.67, P = 0.677). As the distribution of WM scores in the patient group was not Gaussian, a log transformation was performed which did not affect the results. The significant finding for WM in the whole group was not upheld after Simes correction (p_Simes:_ p<0.006). There were no significant group×DMN connectivity interactions in the model of WM (left IPL (F = 0.39, P = 0.679), left PCu (F = 1.72, P = 0.181) and right MFPC (F = 0.16, P = 0.849)). Similarly, with regard to ToM, no significant associations with DMN connectivity were found in whole group analyses (i.e., PCC connectivity with the left PCu (B = -0.69, P = 0.264), left IPL (B = -1.08, P = 0.217), and right MPFC (B = 0.63, P = 0.445)), neither were their significant group×DMN connectivity interactions (left IPL (F = 0.01, P = 0.994), left PCu (F = 0.41, P = 0.665) and right MPFC (F = 0.51, P = 0.600)).

### Exploratory analyses on DMN connectivity and other PANSS symptom dimensions

Exploratory post-hoc multiple linear regression analyses were performed with the remaining 4 symptom clusters (i.e., negative symptoms, disorganization, excitement and emotional distress) (corrected for group, age and sex). In the total group, no associations were found between the remaining symptom clusters and DMN connectivity. In patients, a positive association was found between emotional distress and PCC connectivity with the left IPL (B = 8.41, P = 0.016) and right MPFC (B = 10.44, P = 0.029).

### Environmental exposure and DMN functional connectivity: main and interaction effects

Childhood trauma, cannabis use, and developmental urbanicity were not significantly associated with DMN functional connectivity in the whole group. There were no significant G×E interactions in the model of DMN functional connectivity (Table 4).

**Table 4:** Interactions between environmental risk and group for the regions that are functionally connected to the PCC seed.

	Environmental risk x group interactions					
	Childhood trauma		Cannabis use		Developmental urbanicity	
	F	P	F	p	F	p
Left inferior parietal lobule	0.65	0.525	1.28	0.280	0.80	0.450
Left precuneus	0.25	0.780	0.21	0.812	0.21	0.814
Right medial prefrontal cortex	0.06	0.945	1.19	0.307	1.54	0.218

The F and P-values represent the results of the Wald test. No interactions were significant after Simes correction (P_Simes_<0.006).

### Main effect of AP medication on functional connectivity

There was no significant association between lifetime AP use and functional connectivity between the PCC seed and left IPL (B = 0.00, P = 0.434), left PCu (B = -0.00, P = 0.370) or right MPFC (B = 0.00, P = 0.820).

## Discussion

The objective of the current study was to examine functional connectivity within the DMN in patients with (increased risk for) psychotic disorder. The main finding was that patients and siblings had a similar pattern of increased connectivity between the PCC seed and other regions of the DMN (i.e., left IPL, left PCu and right MPFC) compared to controls. DMN connectivity was not associated with (subclinical) psychotic or cognitive symptoms. The association between familial risk and DMN connectivity was not conditional on environmental exposure.

### DMN connectivity in patients with psychotic disorder

This study adds to the notion of altered interregional functional connectivity in psychotic disorder. Research to date has produced conflicting results as to whether connectivity within the DMN is increased or decreased in schizophrenia. For example, Liu and colleagues have found increased DMN connectivity between the MPFC and parietal regions and between the PCC and temporal regions in schizophrenia during resting-state [24]. Increased DMN connectivity has been described in most of the studies on schizophrenia [19,22,74,75] using either seed-based correlation analysis or ICA. In contrast, a handful of studies have reported decreased DMN connectivity [16,19,76], for example between the PCC and the lateral parietal/medial PFC and PCu [28]. Possible explanations for these inconsistencies may be that the DMN does not comprise a single network, but instead may include several networks, some of which may be altered in schizophrenia [8,77]. The interaction between different networks may lead to a dynamic pattern of dysconnectivity and could contribute to the discrepancy in findings. In addition, conflicting observations across resting-state fMRI studies may also be attributed to several other factors, including differences in patient population, sample size, cohort characteristics, confounding factors (e.g., psychotropic medication), and analytical procedures. Interestingly, the studies reporting decreased DMN connectivity predominantly used ICA analysis [16,19,76], whereas studies demonstrating increased DMN connectivity have used both ICA and seed-based methods [15,19,75]. The two largest studies, one using ICA analysis (n = 258 [16]) and the other seed-based analysis (n = 103 [60]) displayed respectively decreased and increased DMN connectivity. Of note, although univariate (e.g., seed-based correlation) and multivariate (e.g., ICA) statistical analyses may be of influence on the connectivity measurements, a direct comparison of both methods yielded equal results [78,79].

The present study used a relatively large sample size. Nevertheless, the statistical effects were comparatively small, which in combination with adequate statistical power suggests that true DMN effect sizes in psychotic disorder may be less strong than previously stated in smaller studies. However, it has to be noted that a recent study by Khadka et al. (2013) examined the posterior DMN (i.e., cingulate gyrus and PCu) with ICA in a comparably large sample (n = 258) as the current sample and found decreased DMN connectivity. In conclusion, the current evidence indicates that functional integration across regions of the DMN is altered in psychotic disorder although methodological differences across studies preclude definite conclusions. Therefore, using more standardized methods across symptom-based and/or intermediate phenotypes may help to improve the level of evidence.

Regions of the DMN are involved in mental functions such as the responsiveness to salient environmental events, awareness of the environment (IPL), self-referential or introspectively oriented mental activity, decision making (MPFC), self-processing, consciousness and memory processes (PCu) [5,80]. An overactive DMN as found in the current study could mediate distorted boundaries between imagination and perceptions from the external world, and between self and others. Thus, the DMN may underlie formation of psychotic symptoms and social and neurocognitive dysfunction [62,81] (see below).

### DMN connectivity and familial risk for psychotic disorder

The siblings in this study exhibited increased DMN connectivity in similar parts of the DMN network as the patients. Siblings showed intermediate PCC connectivity with the left IPL and left PCu compared to patients and controls, whereas PCC connectivity with the MPFC was slightly higher compared to that of patients. This overlap suggests that DMN connectivity is associated with familial (and possibly genetic) factors. Thus, increased connectivity between the PCC and the left IPL, left PCu and right MPFC may represent trait-related intrinsic network alterations. The current findings replicate other seed-based studies that demonstrated a similar increased DMN functional connectivity ‘intermediate phenotype’ for psychotic disorder [22–24] Contrary to our findings, in two studies there was no evidence for altered connectivity within the DMN of first-degree relatives [16,21], and in one study there was evidence for a decreased DMN functional connectivity ‘intermediate phenotype’ [16]. Resting-state fMRI studies in individuals at ultra-high risk for psychosis [82] and first-episode schizophrenia [75,83] also revealed increased DMN functional connectivity, especially in frontal and parietal regions. Reported findings, in combination with the present results, suggest that DMN abnormalities in patients with psychotic disorder are associated with pre-existing vulnerability and persist over the course of the illness.

### Clinical correlates of altered DMN functional connectivity

There was no significant association between DMN connectivity and positive symptoms in the patients. These results are in line with a recent large study in which correlations with PANSS positive scores did not survive Bonferroni correction [20] and contradictory to previous resting-state fMRI studies that suggest that severity of positive symptoms is associated with either increased [1,22,28] or decreased [28,29] DMN connectivity, depending on the location in the brain (i.e., frontal, parietal or temporal). A possible explanation for the absence of an association between positive symptoms and DMN functional connectivity in the current study is that most patients were in clinical remission, as reflected by relatively low PANSS scores with little variance.

In addition to an absence of associations with positive symptoms, there was also no significant association between WM and DMN connectivity in the present study. An explanation for the absence of an association between DMN connectivity and WM in patients with psychotic disorder may be that the more severe cognitive impairments in schizophrenia are the result of impaired between-network interactions, rather than altered within-network connectivity [21]. To further clarify the “connectivity at rest” (endo)phenotype and its differential relationships with cognitive functioning in patients and siblings, studies on network interactions are warranted.

Increased DMN connectivity was not associated with altered social cognition in individuals with (risk for) psychotic disorder. To our knowledge, only four fMRI studies have investigated the association between social cognition and resting-state DMN connectivity in individuals (at risk of) mental disorder, focusing on psychotic disorder and autism spectrum disorder (ASD). A study in unaffected first-degree relatives of individuals with schizophrenia and healthy controls found that for all participants connectivity between temporal regions and frontal-temporal regions predicted social functioning, empathy and perspective-taking [33]. Studies in ASD showed that social cognitive deficits in ASD were associated with decreased DMN connectivity between the PCC and superior frontal gyrus and between the precuneus and MPFC/anterior cingulate cortex. In addition, social cognitive deficits were associated with increased connectivity between the PCC and the temporal lobe [84–86].

Exploratory post-hoc symptom analyses showed that (altered) DMN connectivity may be associated with emotional distress in patients with psychotic disorder. A task-based fMRI study investigating neural circuits underling emotional distress in healthy individuals found an association between this mental state and brain activation in several regions, including the MPFC and PCC [87]. However, to our knowledge no resting-state fMRI studies have been conducted in patients with psychotic disorder examining the association between emotional distress and DMN functional connectivity. Therefore, future studies are warranted to further investigate this issue.

### Association with environmental exposure

The present study did not provide evidence for a differential impact of environmental exposures on DMN functional connectivity in individuals with (risk) for psychotic disorder. Both familial liability and exposure to environmental risk factors have previously been associated with functional DMN alterations [17,37–40]. However, the studies providing evidence for an association between DMN alterations and environmental factors were conducted in traumatized and healthy populations, whereas no such studies have been carried out in psychotic disorder, let alone studies on GxE. It may be that DMN functional connectivity is not a sensitive enough outcome measure to investigate specific G×E interactions. For example, there is evidence to suggest that these environmental risk factors act through a final common pathway of dopamine (DA) dysregulation in regions of the mesolimbic circuit [88]. Therefore, the functional connectivity in mesocorticolimbic circuits [89] may be a good candidate for future resting-state fMRI research examining this type of interactions.

### Methodological considerations

Advantages of the current study were the large sample size, the use of a representative population of patients with psychotic disorder (and siblings), and the correction for several potential confounding factors. Cannabis and other drugs, as well as alcohol and tobacco may have an effect on brain connectivity [66–70], but previous studies (e.g.,[21,26]) have not always corrected for these confounders and sample sizes were generally much smaller, likely contributing to the variance in study results.

Most of the patients in this study were receiving second generation AP medication at the time of scanning. The effect of AP medication on intrinsic networks is still unclear, although some studies suggest that AP medication normalizes aberrant connectivity [90,91]. However, in the current study there was no main effect of AP on DMN functional connectivity. Furthermore, both medicated patients and non-medicated siblings showed similar patterns of altered connectivity as compared to controls, which argues against this interpretation.

In seed-based analysis, variations in the seed positioning could have impacted the pattern of functional connectivity observed [92]. Nonetheless, the present study has face validity as previous resting-state fMRI studies of smaller sample sizes, and analysed with both seed-based and ICA techniques, have shown increased connectivity in similar regions of the DMN.

The physiological state (i.e., emotional state and arousal level) of the participants was not measured which could lead to altered functional connectivity. That is, studies have shown that a reduced arousal level was associated with reduced functional connectivity during rest [93]. In future studies, it would be valuable to include self-report questionnaires, administered after scanning, on for example wakefulness during the scan session.

The present study comprised cross-sectional data-analyses, precluding any causal and sequential inferences.

## Supporting Information

S1 Table Associations between genetic risk of psychotic disorder (group) and functional connectivity adjusted for additional confounders. (DOCX)

S2 Table Associations between genetic risk of psychotic disorder (group) and functional connectivity without siblings and controls with a history of affective disorder. (DOCX)
