# Supplementary material for: Default Mode Network Connectivity as a Function of Familial and Environmental Risk for Psychotic Disorder
Source: PLoS One. 2015 Mar 19;10(3):e0120030. doi: 10.1371/journal.pone.0120030 (PMC4366233; doi:10.1371/journal.pone.0120030)
Supplement: S1 Table — (DOCX) [file pone.0120030.s001.docx]

**Table S1.** **Associations between genetic risk of psychotic disorder (group) and functional connectivity adjusted for additional confounders**

| **Regions of Interest** | **Functional Connectivity N = 195** | | | **Group differences in functional connectivity** | | | | | |
| --- | --- | --- | --- | --- | --- | --- | --- | --- | --- |
|  | **Patients (n=63)** | **Siblings (n=73)** | **Controls (n=59)** | **P vs. C** | | **S vs. C** | | **P vs. S** | |
|  | **mean (SD)** | **mean (SD)** | **mean (SD)** | **B** | **p** | **B** | **p** | **B** | **p** |
| **Left inferior parietal lobule** | 0.22 (0.21) | 0.23 (0.23) | 0.13 (0.22) | 0.16 | 0.001^*^ | 0.13 | 0.001^*^ | 0.03 | 0.504 |
| **Left precuneus** | 0.37 (0.18) | 0.35 (0.17) | 0.27 (0.14) | 0.12 | 0.000^*^ | 0.10 | 0.001^*^ | 0.02 | 0.488 |
| **Right medial prefrontal cortex** | 0.22 (0.14) | 0.26 (0.15) | 0.15 (0.14) | 0.11 | 0.000^*^ | 0.12 | 0.000^*^ | -0.00 | 0.961 |

The Bs represent the regression coefficients from multiple linear regression analysis in STATA corrected for age, sex, handedness, level of education, tobacco, alcohol, cannabis and other drugs. Abbreviations: P=patients; S=siblings, C=controls; SD=standard deviation; the asterisks (*) represent areas which are significant after Simes correction (P_Simes_<0.033).
