# Supplementary material for: Default Mode Network Connectivity as a Function of Familial and Environmental Risk for Psychotic Disorder
Source: PLoS One. 2015 Mar 19;10(3):e0120030. doi: 10.1371/journal.pone.0120030 (PMC4366233; doi:10.1371/journal.pone.0120030)
Supplement: S2 Table — (DOCX) [file pone.0120030.s002.docx]

**Table S2. Associations between genetic risk of psychotic disorder (group) and functional connectivity without siblings and controls with a history of affective disorder**

| **Regions of Interest** | **Functional Connectivity N = 202** | | | **Group differences in functional connectivity** | | | | | |
| --- | --- | --- | --- | --- | --- | --- | --- | --- | --- |
|  | **Patients (n=73)** | **Siblings (n=67)** | **Controls (n=62)** | **P vs. C** | | **S vs. C** | | **P vs. S** | |
|  | **mean (SD)** | **mean (SD)** | **mean (SD)** | **B** | **p** | **B** | **p** | **B** | **p** |
| **Left inferior parietal lobule** | 0.22 (0.21) | 0.21 (0.22) | 0.14 (0.20) | 0.12 | 0.002^*^ | 0.08 | 0.028^*^ | 0.04 | 0.299 |
| **Left precuneus** | 0.32 (0.16) | 0.28 (0.16) | 0.22 (0.13) | 0.11 | 0.000^*^ | 0.07 | 0.011^*^ | 0.04 | 0.101 |
| **Right medial prefrontal cortex** | 0.23 (0.15) | 0.25 (0.17) | 0.18 (0.15) | 0.08 | 0.008^*^ | 0.09 | 0.002^*^ | -0.01 | 0.725 |

The Bs represent the regression coefficients from multiple linear regression analysis in STATA corrected for age, sex, handedness and level of education. Abbreviations: P=patients; S=siblings, C=controls; SD=standard deviation; the asterisks (*) represent areas which are significant after Simes correction (P_Simes_<0.033).
